# BMP2 and mechanical loading cooperatively regulate immediate early signalling events in the BMP pathway

**DOI:** 10.1186/1741-7007-10-37

**Published:** 2012-04-30

**Authors:** Jessica Kopf, Ansgar Petersen, Georg N Duda, Petra Knaus

**Affiliations:** 1 Institute for Chemistry/Biochemistry, FU Berlin, Berlin, Germany; 2 Julius Wolff Institute, Charité-Universitätsmedizin Berlin, Germany; 3 Berlin-Brandenburg Center for Regenerative Therapies, Berlin, Germany; 4 CMSC, Charité-Universitätsmedizin, Berlin, Germany

## Abstract

**Background:**

Efficient osteogenic differentiation is highly dependent on coordinated signals arising from growth factor signalling and mechanical forces. Bone morphogenetic proteins (BMPs) are secreted proteins that trigger Smad and non-Smad pathways and thereby influence transcriptional and non-transcriptional differentiation cues. Crosstalk at multiple levels allows for promotion or attenuation of signalling intensity and specificity. Similar to BMPs, mechanical stimulation enhances bone formation. However, the molecular mechanism by which mechanical forces crosstalk to biochemical signals is still unclear.

**Results:**

Here, we use a three-dimensional bioreactor system to describe how mechanical forces are integrated into the BMP pathway. Time-dependent phosphorylation of Smad, mitogen-activated protein kinases and Akt in human fetal osteoblasts was investigated under loading and/or BMP2 stimulation conditions. The phosphorylation of R-Smads is increased both in intensity and duration under BMP2 stimulation with concurrent mechanical loading. Interestingly, the synergistic effect of both stimuli on immediate early Smad phosphorylation is reflected in the transcription of only a subset of BMP target genes, while others are differently affected. Together this results in a cooperative regulation of osteogenesis that is guided by both signalling pathways.

**Conclusions:**

Mechanical signals are integrated into the BMP signalling pathway by enhancing immediate early steps within the Smad pathway, independent of autocrine ligand secretion. This suggests a direct crosstalk of both mechanotransduction and BMP signalling, most likely at the level of the cell surface receptors. Furthermore, the crosstalk of both pathways over longer time periods might occur on several signalling levels.

## Background

Despite considerable advances in regenerative medicine and orthopaedic surgery, delayed fracture healing or non-unions of fractures still represent an important clinical concern [1]. Bone morphogenetic proteins (BMPs) are major and indispensable players during bone repair [2,3]. After approval by the Food and Drug Administration in 2001 and 2002, recombinant human BMP2 and recombinant human BMP7 have been used clinically in different applications. However, roughly 1,000 times the normal physiological concentration has to be administered, and in many cases treatment is not superior to autologous bone grafting [4,5]. If BMPs are to be widely used as powerful stimuli, a molecular understanding of their functionality under physiological and diseased conditions appears mandatory.

BMPs belong to the transforming growth factor-β (TGF-β) superfamily and were originally described as being able to induce bone formation. Today, it is known that there are about 25 different BMPs and they are capable of doing much more: they guide many other processes during organ development, tissue homeostasis and repair [6]. However, BMP2, -4, -6, -7 and -9 in particular play pivotal roles in bone morphogenesis [7].

BMP ligands signal by binding to heteromeric complexes of two types of Ser/Thr kinase receptors (BMP type I and type II receptors). Upon ligand binding, intracellular R-Smads (Smad1/5/8) become phosphorylated by BMPRI, followed by trimeric complex formation with Smad4 and subsequent nuclear translocation to regulate BMP target gene transcription. In addition, BMPs are known to activate several non-Smad pathways that involve signalling via mitogen-activated protein kinases (MAPK) (for example, p38, extracellular signal-regulated kinase (Erk) 1/2) or Akt/protein kinase B (PKB) [8]. The combination of both Smad and non-Smad signalling pathways and their respective intensities explain the pleiotropic and cell context specific effects of BMPs.

Each step of the BMP signalling cascade is tightly controlled by antagonists, co-receptors, intracellular associated proteins or by crosstalk to other growth factor pathways [6]. R-Smad molecules in particular constitute signalling platforms to other pathways by multiple post-translational modifications such as phosphorylation or ubiquitination in their linker region [9].

Similar to BMPs, mechanical boundary conditions are crucial for bone development, homeostasis and repair [10]. However, little is known about the impact of mechanical forces on the BMP signalling cascade. Such interaction might be highly relevant since *in vivo *administered recombinant BMPs (rBMPs) seem to be much less potent than *in vitro*. The link between macroscopic bone loading and cellular events is controlled by mechanotransduction pathways, which are still poorly understood. However, the impact of those mechanotransduction pathways on anabolic effects in bone tissue appears indispensable [11]. It is well-known that bone unloading leads to a loss in bone mass [12], or that the rigidity of fracture fixation critically influences the healing outcome [13,14]. The process of mechanotransduction mainly involves three steps: mechanosensing, conversion of mechanical signals into biochemical ones and subsequent signal propagation [15]. Mechanosensing in osteoblasts likely includes multiple pathways involving signalling via integrins, G-protein coupled receptors or ion channels [16,17]. In this context, mechanical signals that control cell fate decisions may comprise active forces, such as loading or shear forces, but may also be encoded by substrate characteristics like stiffness, geometry or ligand spacing [18,19].

Therefore, the aim of this study was to provide further insights into the influence of mechanical forces on BMP signalling. We established a bioreactor system that allows cyclic compressive loading of osteoblast precursor cells in a three-dimensional environment with concurrent BMP2 stimulation. We could show that immediate early BMP signalling events are strongly potentiated by mechanical forces. We conclude that this effect is independent of autocrine BMP ligand secretion and thus gives striking evidence that mechanotransduction pathways directly target BMP signalling molecules without gene expression.

## Results

### Bone morphogenetic protein signalling dynamics in human fetal osteoblasts under two- and three-dimensional culture conditions

**Figure 1:**
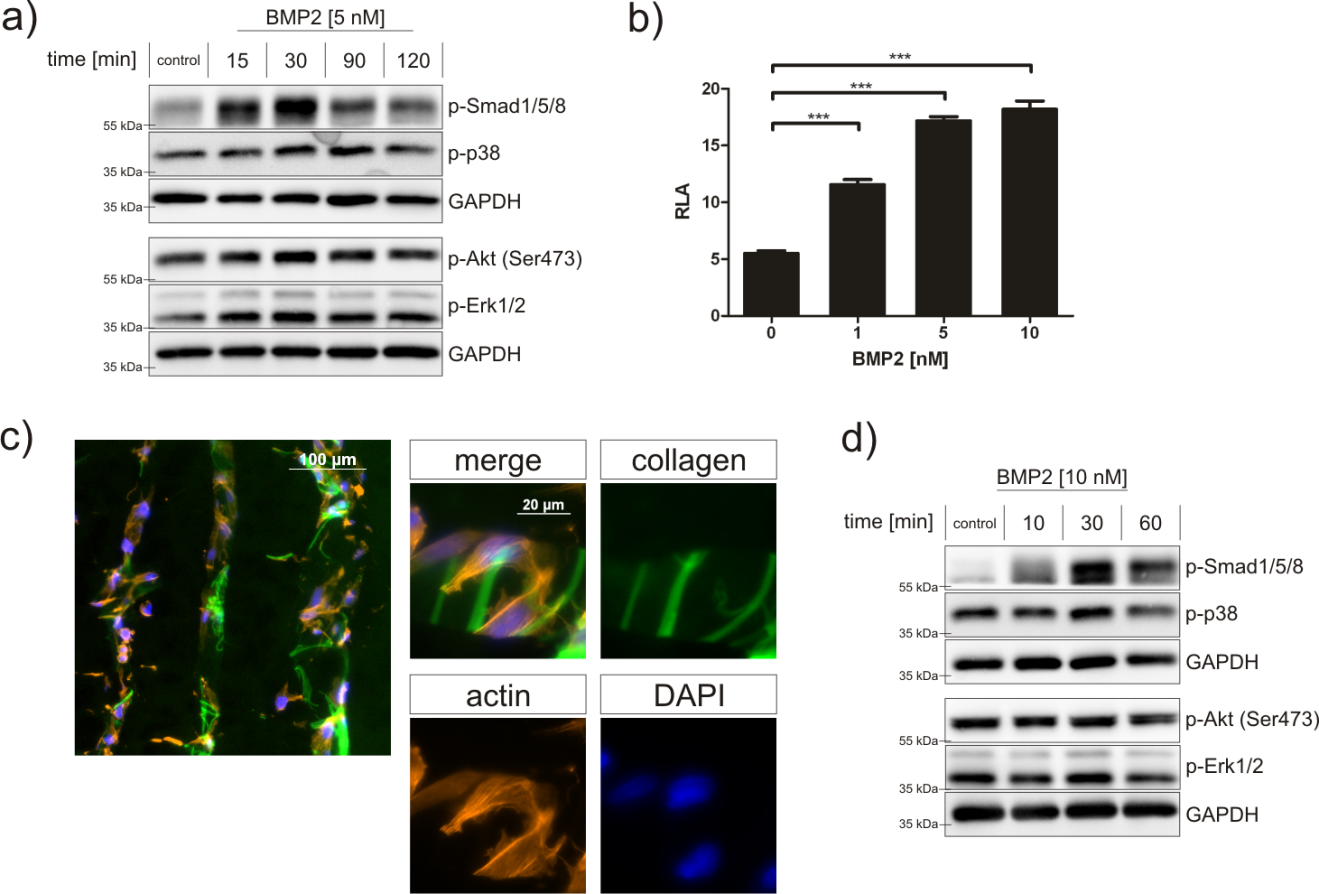
**Bone morphogenetic protein signalling dynamics in hFOBs under two-dimensional and three-dimensional culture conditions**. **(a) **hFOBs in two-dimensional monolayer cultures were stimulated with 5 nM BMP2 for indicated time points and protein phosphorylation was analysed by western blot using specific antibodies. **(b) **hFOBs in two-dimensional monolayer cultures were transfected with a BMP responsive reporter construct (BRE-Luc) and stimulated with different ligand concentrations for 24 hours. Bar charts depict means ± standard error of the mean of relative luciferase activity (RLA); *n *= 3; ***P < 0.001. **(c) **hFOBs were cultured on collagen scaffolds and cell morphology was assessed by immunofluorescent staining. Cell morphology was visualized by actin staining (red), cell nuclei were counterstained by DAPI (blue) and collagen matrix is depicted in green. **(d) **hFOBs were cultured on collagen scaffolds, stimulated with 10 nM BMP2 and protein phosphorylation was analysed by western blot using specific antibodies. BMP: bone morphogenetic protein; DAPI: 4'-6-diamidino-2-phenylindole; hFOB: human fetal osteoblast; RLA: relative luciferase activity.

Mesenchymal precursor cells respond to BMP2 stimulation by inducing Smad and several non-Smad signalling cascades [8]. To investigate the influence of mechanical forces on BMP signalling events, we used the osteoblastic precursor cell line human fetal osteoblasts (hFOBs). Since little is known about BMP signalling in this cell type, we examined the BMP responsiveness of hFOBs under two-dimensional monolayer culture conditions (Figure 1a). BMP2 stimulation strongly induced Smad1/5/8 phosphorylation, which peaked around 30 minutes after stimulation. In addition, non-Smad pathways involving MAPK and Akt/PKB were initiated, as shown by p38, Erk1/2 and Akt phosphorylation. BMP signalling activity was furthermore assessed by a BMP/Smad responsive reporter gene assay (BRE-Luc). Stimulation of BMP2 for 24 hours led to a significant dose-dependent increase in luciferase activity (Figure 1b).

To investigate the influence of mechanical triggers on the BMP signalling cascade, hFOB were seeded on Optimaix^® ^scaffolds (Matricel, Herzogenrath, Germany). To ensure efficient cell growth and adhesion, as well as optimal ligand distribution within the matrix, we analysed cell morphology and signalling dynamics in this culture system (Figure 1c, d). Cells were distributed homogenously throughout the construct, adhered to the collagen scaffold fibres and showed proper cell spreading (Figure 1c). Furthermore, BMP signalling dynamics resembled those under two-dimensional culture conditions (Figure 1d). Smad1/5/8 phosphorylation occurred within 10 minutes of ligand addition, indicating a fast ligand distribution throughout the scaffold due to its macroporous structure. Thus, hFOB cultivation on three-dimensional collagen scaffolds represents a suitable system to further study BMP signalling under concurrent mechanical stimulation.

### Mechanical loading parameter

**Figure 2:**
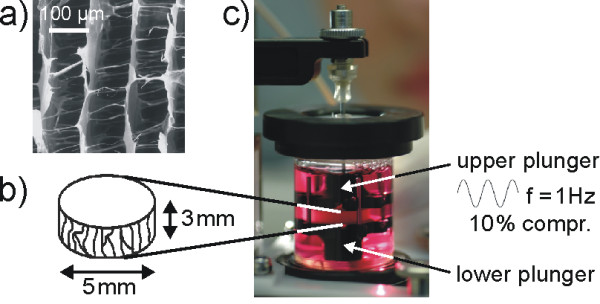
**Mechanical loading parameter**. **(a) **Scanning electron microscopy exposure of collagen scaffolds. **(b) **Schematic representation of mechanical loading setup. Cells were seeded on cylindrical collagen scaffolds and load was applied along the symmetry axis of the scaffold (= pore direction). **(c) **Mechanical loading was performed in a custom-made bioreactor system. Scaffolds were compressed by 10% at a frequency of 1 Hz.

Mechanical forces as well as BMP ligands exert anabolic effects on bone metabolism and both are essential for osteogenic differentiation during bone development and healing [11]. To investigate whether mechanical signals interfere with BMP signalling events, we subjected hFOBs grown on collagen scaffolds to mechanical loading, BMP2 stimulation or a combination of both for up to 24 hours. Figure 2 depicts a rough schematic overview of the mechanical loading device and the most important loading parameters.

### Bone morphogenetic protein 2 and mechanical loading cooperatively regulate immediate early bone morphogenetic protein-induced signalling events

**Figure 3:**
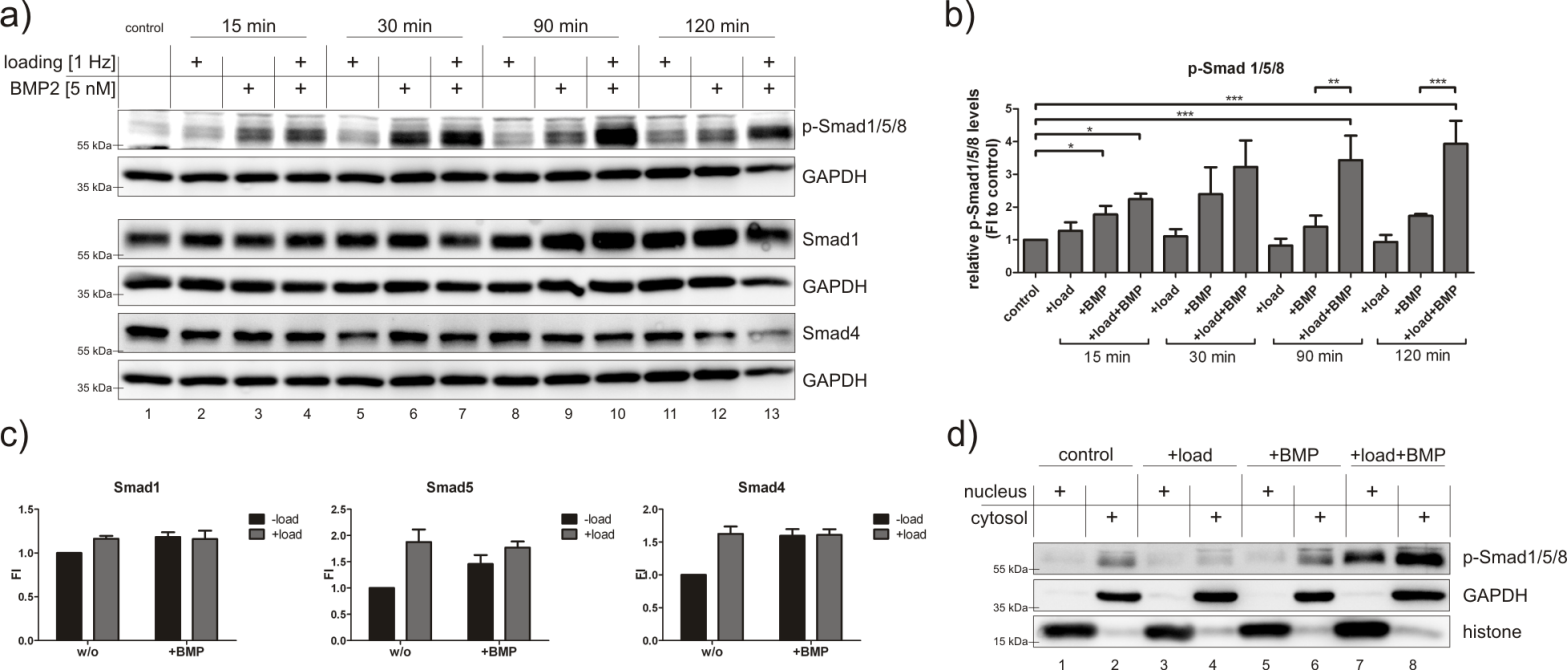
**Bone morphogenetic protein 2 and mechanical loading synergistically regulate bone morphogenetic protein-induced Smad phosphorylation events**. **(a) **hFOBs were seeded on collagen scaffolds and subjected to BMP2 stimulation, mechanical loading or a combination of both. Protein lysates were analysed by western blot using specific antibodies. **(b) **Quantification of western blot analysis depicting phosphoprotein levels normalized to GAPDH. Bar charts represent means ± standard error of the mean (SEM) from three independent experiments; *P < 0.05; **P < 0.01. **(c) **hFOBs were seeded on collagen scaffolds and subjected for 90 minutes to BMP2 stimulation, mechanical loading or a combination of both. Gene expression was analysed by qRT-PCR. Bar charts summarize three independent experiments and depict means ± SEM. **(d) **hFOBs were seeded on collagen scaffolds and subjected for 30 min to BMP2 stimulation, mechanical loading or a combination of both. Nuclear and cytosolic protein lysates were fractionated and analysed by western blot. BMP: bone morphogenetic protein; GAPDH: glyceraldehyde-3-phosphate dehydrogenase; hFOB: human fetal osteoblast; qRT-PCR: quantitative reverse transcriptase polymerase chain reaction; SEM: standard error of the mean.

To analyse whether a direct crosstalk exists between mechanotransduction and BMP signalling cascades, immediate early signalling events downstream of the BMP receptors were investigated. hFOB on collagen scaffolds were subjected for 15, 30, 90 and 120 minutes to BMP2 stimulation, mechanical loading or a combination of both, and Smad1/5/8 phosphorylation was analysed (Figure 3a and 3b). Already after 15 minutes, Smad1/5/8 was phosphorylated when stimulated with BMP2. The phosphorylation peaked after 30 minutes and declined afterwards. When cells were concurrently mechanically loaded, Smad1/5/8 phosphorylation was slightly enhanced 15 and 30 minutes after stimulation. Even more striking, p-Smad1/5/8 levels did not decline after 30 minutes but remained on the same level over up to 120 minutes of stimulation (Figure 3a; lanes 4, 7, 10 and 13). Thus, after 90 and 120 minutes of stimulation p-Smad1/5/8 levels were significantly higher than in samples treated with BMP alone (Figure 3b). In line with that, stronger Smad phosphorylation under concurrent stimulation was also observed after 60 minutes and persisted until 240 minutes of stimulation (Additional file 1). Mechanical loading alone did not cause Smad1/5/8 activation. At the same time, Smad2 phosphorylation was neither affected by BMP2 stimulation nor by mechanical loading or a combination of both (Additional file 2). Total Smad1 and Smad4 protein levels were not altered by the different treatments. This was further sustained by expression analysis of Smad1, -5 and -4 (Figure 3c).

Activation of Smad molecules through C-terminal phosphorylation triggers their nuclear translocation followed by target gene regulation [20]. To examine nuclear shuttling dynamics of Smad1/5/8, cells were stimulated for 30 minutes, nuclear and cytosolic proteins were separated and p-Smad1/5/8 levels were analysed in each fraction. In BMP2-stimulated samples with concurrent mechanical loading, we detected not only stronger phosphorylation of Smad1/5/8 but also an increased nuclear localization of p-Smad1/5/8 (Figure 3d; compare lanes 5 and 7). Taken together, this shows for the first time that mechanical loading promotes both Smad1/5/8 phosphorylation and their subsequent nuclear translocation.

**Figure 4:**
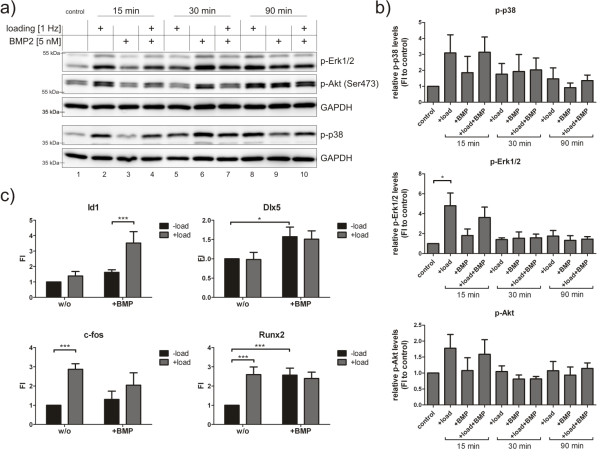
**Bone morphogenetic protein 2 and mechanical loading both regulate early bone morphogenetic protein signalling events**. **(a) **hFOB were seeded on collagen scaffolds and subjected to BMP2 stimulation, mechanical loading or a combination of both. Protein lysates were analysed by western blot using specific antibodies. **(b) **Quantification of western blot analysis depicting phosphoprotein levels normalized to GAPDH. Bar charts represent means ± SEM from three independent experiments; *P < 0.05. **(c) **hFOBs were seeded on collagen scaffolds and subjected for 90 minutes to BMP2 stimulation, mechanical loading or a combination of both. Gene expression was analysed by qRT-PCR. Bar charts summarize four independent experiments and depict means ± SEM; *P < 0.05; **P < 0.01. BMP: bone morphogenetic protein; GAPDH: glyceraldehyde-3-phosphate dehydrogenase; hFOB: human fetal osteoblast; qRT-PCR: quantitative reverse transcriptase polymerase chain reaction; SEM: standard error of the mean.

Signalling via p38, Erk1/2 or Akt is part of BMP-induced non-Smad signalling cascades, and is furthermore involved in mechanotransduction. To investigate the capacity of BMP2 and mechanical load to activate these pathways, hFOBs were treated for up to 90 minutes with BMP2, mechanical load or a combination of both (Figure 4a and 4b). Western blot analysis revealed that, after 15 minutes, p38, Erk1/2 and Akt had already become phosphorylated under loading conditions (Figure 4a; lanes 2 and 4). Phosphorylation of p38, Erk1/2 and Akt by BMP2 showed the strongest induction around 30 minutes of stimulation and declined afterwards. However, no synergistic effect of mechanical loading and BMP2 stimulation was detected on non-Smad signalling cascades. In general, non-Smad signalling dynamics vary between experiments due to the complexity of the system, that is, a three-dimensional culture combined with biochemical versus mechanical stimulation.

After 90 minutes of stimulation, the expression of early BMP and mechanoresponsive target genes was analysed (Figure 4c). Again, cells were stimulated with BMP2, mechanical loading or a combination of the two. *Inhibitor of differentiation 1 *(*Id1*) is one of the earliest BMP target genes, because phosphorylated Smads directly bind to the *Id1 *promoter [21]. *Id1 *expression was slightly induced by BMP2 stimulation after 90 minutes. Surprisingly, this induction was strongly enhanced when cells were concurrently mechanically loaded (induction of 2.8-fold and 7.7-fold). In contrast, *c-fos*, a well-known mechanoresponsive gene [22], was up-regulated by mechanical loading, while BMP2 had no effect on its expression. This finding is in line with the strong Erk1/2 activation by mechanical loading (Figure 4a), which is known to be upstream of *c-fos *gene expression [23,24]. The contrary case was true for *Dlx5*: both under loading and non-loading conditions, BMP2 led to enhanced gene expression. For *Runx2*, BMP2 stimulation, mechanical load and a combination of both resulted in a similar mRNA induction when compared to the control group.

Based on these observations we conclude that BMP-induced Smad1/5/8 signalling is potentiated by mechanical loading. As this effect was already prominent after 15 minutes of stimulation we conclude that this mechanism does not involve autocrine ligand secretion. In addition, mechanical forces and BMP2 synergistically regulate transcription of the early BMP target gene *Id1*.

### Bone morphogenetic protein target gene expression is differentially regulated by bone morphogenetic protein 2 and mechanical loading

To further understand the impact of mechanical forces on BMP signalling outcome towards later time points, cells were stimulated for 24 hours and the gene expression of several BMP target genes as well as of BMP ligands and antagonists was analysed.

**Figure 5:**
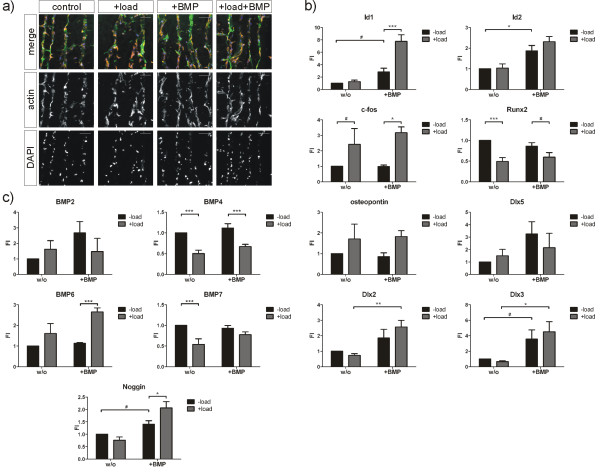
**Bone morphogenetic protein target gene expression in differentially regulated by bone morphogenetic protein 2 and mechanical loading**. **(a) **hFOBs were seeded on collagen scaffolds and subjected for 24 hours to BMP2 stimulation, mechanical loading or a combination of both. Cell morphology was visualized by actin staining (red), cell nuclei were counterstained by DAPI (blue) and collagen matrix is shown in green. Scale bars, 100 μm. **(b and c) **hFOBs were seeded on collagen scaffolds and subjected for 24 hours to BMP2 stimulation, mechanical loading or a combination of both. Gene expression was analysed by qRT-PCR. Bar charts summarize three independent experiments and depict means ± SEM; ^#^*P *≤0.1, *P < 0.05, **P < 0.01, ***P < 0.005. BMP: bone morphogenetic protein; DAPI: 4'-6-diamidino-2-phenylindole; hFOB: human fetal osteoblast; qRT-PCR: quantitative reverse transcriptase polymerase chain reaction; SEM: standard error of the mean.

Scaffolds were subjected to BMP2 stimulation, mechanical loading or a combination of both. After 24 hours, no difference in cell number, morphology or cellular distribution throughout the scaffold between the individual treatments was observed (Figure 5a). Under all conditions, cells were homogenously adhering to collagen fibres and distributed evenly throughout the scaffold. In addition, no significant alterations of the scaffold structure under mechanical loading became evident (Figure 5a). This ensured that the cellular environment remained consistent over the observation time period of up to 24 hours.

Analysing the expression of different BMP target genes after 24 hours revealed that mRNA levels were differentially affected by BMP stimulation and by mechanical loading (Figure 5b). *Id1 *expression was induced by BMP treatment and this induction was significantly enhanced when cells were concurrently loaded. In contrast, *Id2 *expression was also induced by BMP2 but the enhancing effect of mechanical loading was not present. *c-fos *and *osteopontin *expression was strongly up-regulated by mechanical loading, while BMP treatment exhibited no effect. By contrast, *Runx2 *expression, that was induced after 90 minutes (Figure 4c), was down-regulated by mechanical loading after 24 hours. Gene expression of members of the Distal-less homeobox family, *Dlx2 *and *Dlx3*, was induced by BMP2 but expression was not significantly enhanced by concurrent mechanical loading. *Dlx5 *expression after 24 hours of stimulation was not regulated by the different treatments.

To further elucidate the involvement of possible feed-forward regulations by autocrine ligand secretion, we also analysed the expression of *BMP2, -4, -6 *and -*7 *as well as the expression of the BMP antagonist *Noggin *(Figure 5c). All analysed BMP ligands are capable of inducing bone formation; however, they differ in their receptor usage and susceptibility to the antagonist Noggin [25,26]. As expected, *BMP2 *expression was induced by BMP2 stimulation, but general *BMP2 *expression levels were quite weak. Interestingly *BMP4 *and -*7 *were down-regulated by mechanical loading, while expression of *BMP6 *was up-regulated. At the same time, *Noggin *expression was induced by BMP2 stimulation and this was further enhanced by concurrent mechanical loading.

These results demonstrate that mechanical loading directly affects immediate early BMP signalling events. At the same time, BMP and mechanical forces differentially regulate transcription of osteogenic marker genes. Towards later time points, BMP signalling might be indirectly affected through differential expression of BMP ligands and their antagonists. We conclude that BMP signalling is guided by a balanced availability of ligands and antagonists, but also by physical triggers. This highlights the importance of the biomechanical environment for BMP-induced cellular processes, such as differentiation.

## Discussion

The application of recombinant BMPs to foster bone healing has turned out to be less potent than expected from *in vitro *studies. Effective delivery and high doses have been the most limiting factors for clinical treatments combined with the risk of side-effects [4]. There is great emphasis in the field to lower the concentrations of BMPs by approving delivery systems, increasing BMP's potency and, most of all, by understanding the molecular mechanism of supporting crosstalk pathways.

BMP signalling is a tightly controlled cascade that is regulated on different levels ranging from extracellular antagonists to receptor composition and intracellular interacting molecules [6]. On the tissue level, there exists strong evidence that BMP signalling and mechanical forces together regulate bone healing. However, little is known about the molecular mechanism of how mechanical boundary conditions might regulate BMP signalling. A better understanding of the crosstalk between both pathways seems essential to unravel their physiological interaction and to help to gain a better understanding towards an adequate use of both stimuli to improve patient treatment strategies.

In recent years, multiple studies have shown the importance of mechanical forces for cellular differentiation [27,28]. But many *in vitro *studies focusing on osteogenic differentiation were performed in two-dimensional culture systems and few of them on a molecular basis. To better mimic the *in vivo *cellular environment, a three-dimensional culture system is indispensable. Therefore, in this study we investigated early events during osteoblastic differentiation induced by BMP2 under mechanical loading in a three-dimensional environment. hFOBs were seeded on open porous collagen scaffolds (average bulk stiffness of 8.5 ± 0.9 kPa) and mechanically loaded with up to 10% straining. hFOBs properly adhered to collagen fibres, and collagen scaffolds exhibited a suitable and physiological stiffness range for initial osteoblastic differentiation [27]. These cells further showed similar signalling dynamics in three-dimensional when compared to two-dimensional monolayer cultures (Figure 1). The bioreactor setup is tuned to mimic the early phase of bone healing events during tissue formation, keeping culture conditions, oxygen supply and mechanical loading parameters constant [29].

To unravel the molecular mechanism comprising this crosstalk, we analysed BMP-induced signalling at different time points. We investigated early phosphorylation events directly downstream of the activated BMP receptors as well as transcriptional responses at different time points (early and late).

We found that BMP2 stimulation and mechanical load synergistically regulate immediate early phosphorylation events in the BMP pathway (Figure 3). BMP2 stimulation with concurrent mechanical loading resulted in the strongly enhanced C-terminal phosphorylation of Smad1/5/8 followed by an increased nuclear translocation when compared to cells stimulated with BMP2 only. This effect was observed as early as 15 minutes after stimulation and was maintained up to several hours (Figure 3 and Additional file 1).

Based on these findings, we postulate that mechanical signals directly influence immediate early BMP signalling events without the involvement of autocrine ligand secretion. The fact, that loading alone did not show significant differences in Smad1/5/8 phosphorylation or *Id1 *expression further proves this hypothesis. This is in contrast to previous studies where mechanical load was reported to activate the BMP pathway [30,31]. This may be explained by different types of mechanical forces and study design that included pre-cultivation on scaffold matrices prior to loading for up to 7 days. In this case, BMP pathway activation by mechanical loading might be due to autocrine ligand secretion during culture. In fact, Wang *et al*. demonstrated that Noggin addition during mechanical stimulation abolished BMP pathway activation induced by mechanical loading [32].

The first step during mechanotransduction comprises the sensing of extracellular mechanical signals by a mechanoreceptor, such as integrins or ion channels [16]. Especially integrins crosstalk to TGFβ and BMP signalling pathways [33]. Similarly there exists increasing evidence that integrin expression and signalling is also important for BMP-induced signalling during osteogenic differentiation [34-36]. It was demonstrated that both BMP type I and type II receptors co-localize with αvβ integrins [34]. Furthermore, many proteins associated with integrin signalling complexes, such as c-Src or Rack1, are also interacting with the cytoplasmatic tail domain of the BMP type II receptor [37,38]. We hypothesise that integrin activation under loading conditions might lead to altered conformational changes of BMP receptors, which modulate their interactome and alter their signalling properties. Recently, it has been shown that endocytosis of integrin receptors depends on extracellular matrix stiffness and that this altered endocytosis also affects BMP receptor endocytosis and signalling [39]. The route of BMP receptor endocytosis itself critically determines the signalling outcome [40]. We have previously shown that blocking endocytosis inhibits BMP-induced *Id1 *expression while having no effect on *Id2 *[41]. Similarly, mechanical load enhanced BMP-induced expression of *Id1 *but not *Id2 *(Figure 5). Since receptor endocytosis is strongly related to the membrane lipid composition, it is likely that membrane raft microdomains may play an important role as mechanosensing platforms.

Chang *et al*. proposed that integrins might mediate Smad activation under shear stress conditions [42]. In our system, ligand independent Smad1/5/8 activation (that is, C-terminal phosphorylation) was not observed as indicated by load-only treatment (Figure 3). However, ligand independent integrin mediated signalling might be involved in the activation of non-Smad pathways and their target genes.

After 15 minutes of stimulation, mechanical loading led to the strong induction of p38, Akt and Erk1/2 phosphorylation (Figure 4). Erk1/2 and p38 have, in particular, been described as important players during mechanotransduction in mesenchymal precursor cells [23,43,44]. Furthermore, signalling pathways *via *MAPK might be involved in regulating Smad signal intensity and duration. The Smad1 linker region comprises several sequential phosphorylation sites for cyclin dependent kinases (CDKs), MAPK and glycogen synthase kinase three beta (GSK3β) that regulate their transcriptional capacity and prime Smad molecules for degradation via the ubiquitin proteasome pathway [9,45]. In contrast to the Smad pathway, we did not observe synergistic effects of mechanical load and BMP2 on the non-Smad target proteins. But gene regulation under loading conditions of osteogenic marker genes likely involves the interplay of Smad and non-Smad pathways.

Following the BMP pathway further downstream, we analysed the transcriptional regulation of several BMP target genes. Earlier studies tried to elucidate gene expression profiles in osteoblast precursor cells under mechanical stress [46,47]. It was postulated that mechanical load induces osteogenic differentiation [48] and that mechanical forces exert synergistic effects on osteogenic differentiation together with BMP2 [49]. However, these studies are hardly comparable due to different cellular systems, including osteogenic and non-osteogenic cell types, and mechanical stimulation devices in two dimensions and three dimensions. In addition, most studies focused on long-term differentiation events that potentially include feedback signalling loops.

We showed that the transcriptional network mediating early osteogenic differentiation events includes genes regulated by mechanical forces or the BMP ligand only, as well as genes that are synergistically affected by both triggers. This reflects multiple levels of potential crosstalk between the BMP and mechanotransduction pathway. BMP2 stimulation with concurrent mechanical loading led to synergistic regulation of the early BMP target gene *Id1*, a key regulator in BMP-induced osteoblastic differentiation (Figures 3 and 5). We also confirmed this in primary human mesenchymal stem cells (Additional file 3). This is of particular interest, because *Id1 *transcription is not only under the control of Smads but also of early growth response protein one (Egr-1), a transcription factor rapidly induced by mechanical stress [50]. *c-fos*, known to be a major target of mechanotransduction [22], was strongly induced by mechanical loading, while BMP treatment had negligible effects (Figures 3 and 5). However, Smad4 was shown to interact with c-fos, which modulates activating protein one (AP-1) activity [51]. Whether different strain amplitudes trigger different responses or whether there exists a certain strain threshold remains to be elucidated.

Autoregulation of BMP ligand or antagonist expression is one possibility to modulate the signalling pathway endogenously. It has been shown that mechanical loading of osteoblasts leads to a transcriptional up-regulation of several BMP ligands, such as *BMP2, -4, -6*, and -*7 *[32,52-54]. We instead found that different BMP ligand subtypes are differentially affected by loading. While *BMP4 *and *BMP7 *tend to be down-regulated under loading conditions, *BMP6 *expression was positively affected by mechanical loading, even more so when BMP2 was present (Figure 5). These findings are in line with *in vivo *data obtained during fracture healing and distraction osteogenesis [55,56]. Different BMP ligands not only exhibit a distinct spatiotemporal expression pattern but also respond differently to mechanical forces. Interestingly, BMP4 and -6 also differ in their susceptibility to the BMP inhibitor Noggin, with BMP6 being not inhibited by this antagonist [25]. Expression analysis revealed that *Noggin *mRNA was significantly up-regulated by BMP2 and this up-regulation was further enhanced by mechanical loading. Thus *Noggin *regulation is a crucial event during osteogenic differentiation to balance signalling intensity and is also sensitive to mechanical stimulation. Also other TGFβ -superfamily antagonists, such as sclerostin, gremlin and follistatin, are regulated by mechanical forces [53,57,58]. The BMP antagonists may represent an important target to improve bone healing when inter-related to adequate mechanical boundary conditions. Furthermore, other growth factor pathways, such as Wnt or insulin-like growth factor (IGF) signalling, are influenced by mechanical loading. They share many downstream partners and target genes with the BMP pathway and might be also involved in BMP pathway regulation [16].

## Conclusions

**Figure 6:**
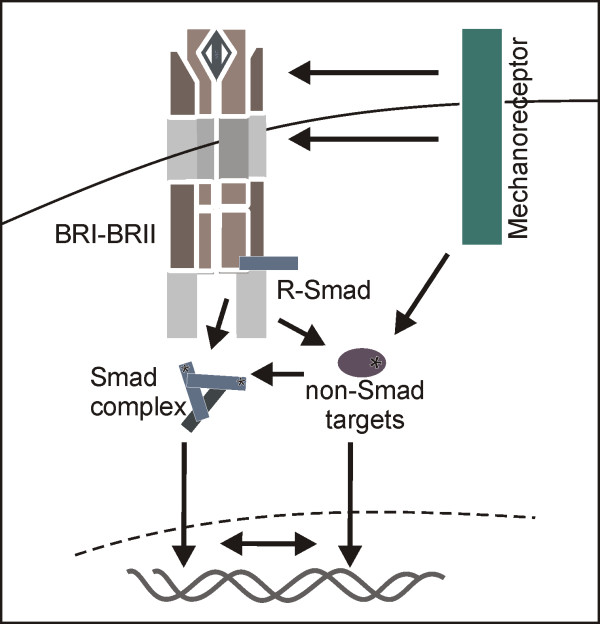
**Crosstalk between bone morphogenetic protein and mechanotransduction pathways might occur on several signalling levels**. Schematic model of possible crosstalk levels between mechanical triggers and the BMP signalling cascade as indicated by arrows. BMP: bone morphogenetic protein; BRI-II: bone morphogenetic protein receptor I or II.

This study highlights the complex interaction of mechanical forces with the BMP signalling cascade. We demonstrated that BMP signalling is directly regulated by mechanotransduction pathways, without the involvement of autocrine ligand secretion. We also gave evidence that crosstalk of both pathways over longer time periods might occur on several signalling levels. A hypothetical model on the interplay between both pathways has been proposed (Figure 6). Direct crosstalk is possible as early as at the receptor level at the plasma membrane, in the cytosol or in the nucleus by altering transcription factor properties. Finally, mechanosensing by inner nuclear membrane proteins, which have been shown to also anchor Smad proteins, may participate in this relationship [59-61].

Although the fine-tuned course of osteogenic differentiation during bone healing still remains unclear, the present work is the first to illustrate the tight interaction of BMP- and mechanical stimuli-associated signalling cascades. These cascades are spatiotemporally highly balanced and are fine-tuned processes that need further analyses for a deeper understanding of their interplay. The general principles, however, have been illustrated and are discussed in the present work. To transfer that knowledge into improvements in bone healing, such as the required stiffness of bone fixations in BMP-treated cases, requires further *in vivo *analyses and *in vitro *characterization. Such knowledge will ultimately help to improve treatments in the majority of clinical cases and, thus, avoid failures of BMP-initiated stimulation of healing.

## Materials and methods

### Cell culture and reagents

hFOB 1.19 (ATCC, Manassas, Virginia, USA) cells were cultured in a 1:1 mixture of Dulbecco's Modified Eagle Medium and Ham's F12 Medium (Invitrogen, Carlsbad CA, USA) supplemented with 10% fetal calf serum (FCS) (Biochrom AG, Berlin, Germany), penicillin (50 units/mL)/streptomycin (5 μg/mL) (PAA, Coelbe, Germany) and 0.3 mg/mL G418 (Invitrogen). Cells were grown under a permissive temperature of 34°C. For loading experiments, cells were seeded on macroporous Optimaix^® ^collagen-I scaffolds (Matricel) at a density of 3.2×10^5 ^cells/scaffold (cylindrical shape of the scaffold; diameter 5 mm, height 3 mm). Scaffold mean pore size was 84 μm as analysed by the manufacturer. Cells were maintained in static scaffold culture for two days prior to any experiment. After transferring scaffolds to the bioreactor system, cells were starved for 3 hours. All experiments were performed under serum starvation conditions to exclude signalling effects by growth factors being present in the FCS. For stimulation of up to 2 hours duration, medium containing 0% FCS was used. For 24 hours stimulation, medium was supplemented with 1% FCS for optimal cell survival. In the bioreactors, cells were mechanically loaded, stimulated with 5 nM BMP2 or treated with a combination of both.

### Mechanical loading parameters

Cyclic axial compressive loading was performed using a custom-made bioreactor system described by Petersen *et al*. [29], which is briefly described as follows. Because axial inter-fragmentary movement was shown to be the main straining component in animal osteotomy models with external fixators, these loading conditions were realized in the bioreactor [62,63]. The compression magnitude was chosen to mimic the mean strain distribution in the fracture gap of a sheep osteotomy model that is known to achieve successful healing within 9 weeks after osteotomy [64]. The selected frequency represents the time pattern of loading during walking and the sine wave is a simplified load pattern based on data gained from patients with instrumented hip implants [65,66]. In detail, cyclic axial compression along the scaffold pore orientation was applied in a sine wave form with a frequency of 1 Hz and a magnitude of 10% scaffold height (= 300 μm). Three hours prior to stimulation, cell-seeded scaffolds were transferred to the bioreactor device. All scaffolds, also non-loaded controls, were positioned between the lower and upper plunger and a small preloading force of 5 mN per scaffolds was adjusted. After 3 hours of starvation, loading and/or BMP stimulation were initiated. Since collagen scaffolds may deform slightly over time, readjustment of scaffold position was conducted for long-term stimulations of 24 hours. The preloading force for each scaffold was automatically readjusted by a positional change of the lower plunger after 2, 4, 8, 12, 16 and 20 hours. Details of the loading protocol are given in Additional file 4.

### Antibodies and western blotting

Protein lysates were subjected to SDS-PAGE and transferred on nitrocellulose membranes by western blot. Membranes were blocked for 1 hour in 3% dry milk powder and incubated with the indicated primary antibodies overnight at 4°C following manufacturer's instructions. The following antibodies were used: glyceraldehyde-3-phosphate dehydrogenase (GAPDH; #2118, Cell Signaling, Danvers, MA, USA), phosphorylated-Smad1/5/8 (#9511, Cell Signaling), total Smad1 (#9743, Cell Signaling), total Smad4 (sc-7966, Santa Cruz), phosphorylated-p38 Thr180/Tyr182 (#V1211, Promega), phosphorylated-ERK 1/2 (pp42/p44 MAPK Thr202/Thy204, #9101, Cell Signaling), phosphorylated-Akt Ser473 (#4051, Cell Signaling) and histone (#9715, Cell Signaling). To guarantee highly quantitative western blots, we avoided stripping the membranes and applied lysates on several gels. Each blot was separately probed for proper loading visualized by GAPDH. Western blot images were quantified using BioProfile Bio1D software (Vilber Lourmat, Eberhardzell, Germany).

### Dual luciferase assay

Cells were transfected with a BMP responsive reporter construct, BRE-Luc [67], using Lipofectamine2000^® ^reagent (Invitrogen). As internal control, a constitutively expressed construct, encoding for *Renilla *luciferase, was co-transfected. Cells were starved for 3 hours in culture medium containing 0.5% FCS and stimulated for 24 hours with different concentrations of BMP2. Cells were lysed in 1× passive lysis buffer (Promega, Madison, WI, USA) and firefly and *Renilla *luciferase activity was measured. Firefly values were normalized to the internal control and firefly/*Renilla *ratios are depicted as relative luciferase activity.

### Nuclear and cytoplasmic protein fractionation

Nuclear and cytosolic protein extracts were generated using ProteoJET^® ^Cytoplasmic and Nuclear Protein Extraction Kit (Fermentas, Helsinki, Finland). Isolation was done according to the manufacturer's protocol. In order to prevent protein degradation and dephosphorylation, all buffers were supplemented with 1× Complete^® ^protease inhibitor cocktail (Roche, Penzberg, Germany) and 50 mM sodium fluoride.

### Quantitative real-time PCR

Total RNA was isolated using NucleoSpin^® ^isolation kit (Macherey&Nagel, Dueren, Germany) and 1 μg of RNA was subjected to reverse transcription. For all used primers, amplification efficiencies were determined and mean normalized expression ratios, using *HPRT *as the reference gene, were calculated using the ΔΔc_T _method with efficiency correction. Primer sequences as well as gene accession numbers are depicted in Additional file 5. Constant expression of the house-keeping gene *HPRT *was validated by geNorm software (Center for Medical Genetics, Ghent University Hospital, Ghent, Belgium) (Additional file 6).

### Immunofluorescent staining

For immunofluorescent staining, collagen scaffolds were fixed with 4% paraformaldehyde (PFA), quenched in 50 mM ammonium chloride and subsequently transferred in 5% warm gelatine solution. Doing so, the sample's geometry was stabilized by gelatine gelation at 4°C. Scaffolds were then embedded in Tissue-Tek^® ^O.C.T. Compound (Sakura, Alphen aan den Rijn, Netherlands) and 25 μm cryosections were cut. Sections were fixed again with 4% PFA for 5 minutes and the actin cytoskeleton was visualized by Phalloidin-Alexa594 (Invitrogen). Staining of nuclei was performed by 4'-6-diamidino-2-phenylindole. Collagen structures are depicted by their autofluorescent properties in the HE38 filter set (Zeiss, Jena, Germany) with an excitation of 470/40 nm and an emission of 525/50 nm. Images were acquired by epifluorescence microscopy (Zeiss Axiovert 200 M).

### Statistical analysis

Comparison of multiple groups was done by one-way or two-way analysis of variance (ANOVA) with Bonferroni multiple comparison post-test analysis for one-way ANOVA and Sidak-Holm multiple comparison post-test analysis for two-way ANOVA. Statistical calculations were performed using SigmaPlot software (Systat Software Inc., Chicago, USA) and a *P*-value below 0.05 was considered statistically significant.

## Competing interests

The authors declare that they have no competing interests.

## Authors' contributions

JK carried out all biochemical and cell biology experiments, made a substantial contribution to the conception and design as well as data analysis and drafted the manuscript. AP contributed to the conception and design, was responsible for technical optimisation of the mechanical loading parameters and critically revised the manuscript. GND conceived the study, helped in data interpretation, provided a clinical point of view and participated in critical manuscript revision. PK conceived the study, and helped in data interpretation, and drafting and revising the manuscript. All authors read and approved the final manuscript.

## Supplementary Material

Additional file 1 **BMP2 and mechanical loading synergistically regulate BMP-induced Smad phosphorylation events**. **(a and b) **hFOBs were seeded on collagen scaffolds and subjected to BMP2 stimulation, mechanical loading or a combination of both. Protein lysates were analysed by western blot using specific antibodies. Click here for file

Additional file 2 **Smad2 is not phosphorylated by BMP2 stimulation, mechanical loading or a combination of both**. hFOBs were seeded on collagen scaffolds and subjected to BMP2 stimulation, mechanical loading or a combination of both for indicated time points. As positive control, hFOBs were stimulated for 30 minutes with 100 pM TGF-β1. Protein lysates were analysed by western blot using specific antibodies. Click here for file

Additional file 3 **BMP2 and mechanical load synergistically regulate *Id1 *gene expression in primary human mesenchymal stem cells**. Data of one representative experiment is depicted. Human primary mesenchymal stem cells (hMSCs) were embedded in fibrin gels and loaded for 3 days in the absence or presence of 10 nM BMP2. Embedding and loading was performed as described previously [68]. Total RNA was extracted and *Id1 *gene expression was analysed by qRT-PCR. Click here for file

Additional file 4 **Mechanical loading protocol**. Running protocol for short-term (up to 120 minutes) and long-term (up to 24 hours) mechanical loading. Click here for file

Additional file 5 **Primer sequences**. Sequence of primers used for qRT-PCR. Click here for file

Additional file 6 **Validation of a reference gene for qRT-PCR**. Validation of *HPRT *as house-keeping reference gene using geNorm software [69]. Click here for file
