# Supplementary figures and images for: BMP2 and mechanical loading cooperatively regulate immediate early signalling events in the BMP pathway
Source: BMC Biol. 2012 Apr 30;10:37. doi: 10.1186/1741-7007-10-37 (PMC3361481; doi:10.1186/1741-7007-10-37)

a)

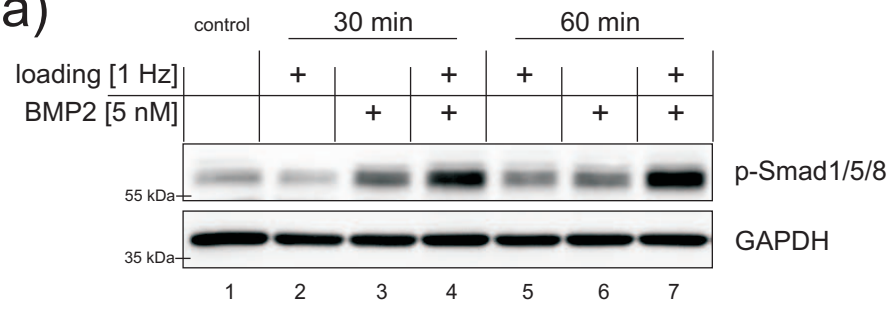

b)

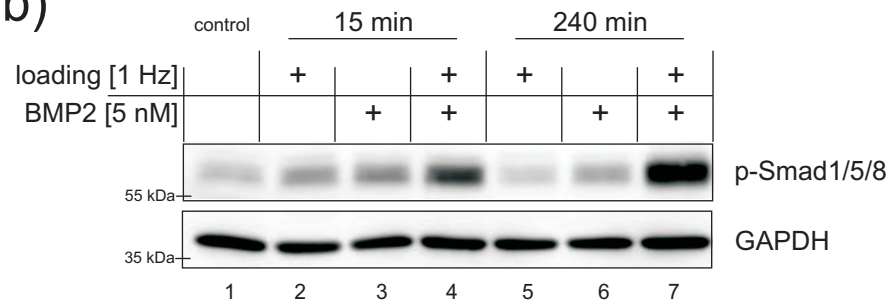

Supplement: Additional file 1 — BMP2 and mechanical loading synergistically regulate BMP-induced Smad phosphorylation events. (a and b) hFOBs were seeded on collagen scaffolds and subjected to BMP2 stimulation, mechanical loading or a combination of both. Protein lysates were analysed by western blot using specific antibodies. [file 1741-7007-10-37-S1.PDF]

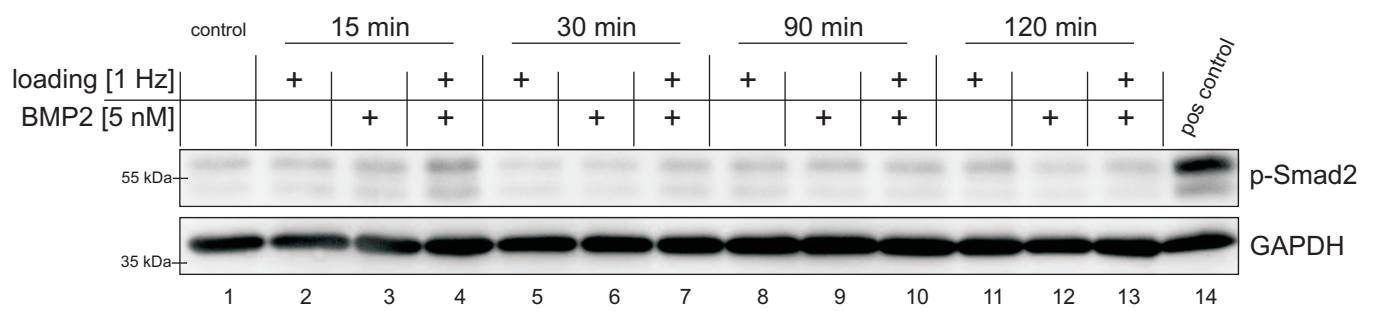

Supplement: Additional file 2 — Smad2 is not phosphorylated by BMP2 stimulation, mechanical loading or a combination of both. hFOBs were seeded on collagen scaffolds and subjected to BMP2 stimulation, mechanical loading or a combination of both for indicated time points. As positive control, hFOBs were stimulated for 30 minutes with 100 pM TGF-β1. Protein lysates were analysed by western blot using specific antibodies. [file 1741-7007-10-37-S2.PDF]

# Id1

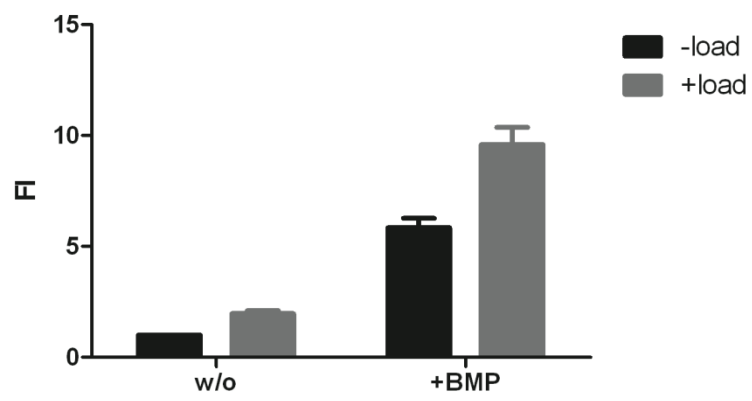

Supplement: Additional file 3 — BMP2 and mechanical load synergistically regulate Id1 gene expression in primary human mesenchymal stem cells. Data of one representative experiment is depicted. Human primary mesenchymal stem cells (hMSCs) were embedded in fibrin gels and loaded for 3 days in the absence or presence of 10 nM BMP2. Embedding and loading was performed as described previously [68]. Total RNA was extracted and Id1 gene expression was analysed by qRT-PCR. [file 1741-7007-10-37-S3.PDF]
