# Supplementary material for: BMP2 and mechanical loading cooperatively regulate immediate early signalling events in the BMP pathway
Source: BMC Biol. 2012 Apr 30;10:37. doi: 10.1186/1741-7007-10-37 (PMC3361481; doi:10.1186/1741-7007-10-37)
Supplement: Additional file 4 — Mechanical loading protocol. Running protocol for short-term (up to 120 minutes) and long-term (up to 24 hours) mechanical loading. [file 1741-7007-10-37-S4.PDF]

	protocol						
**action**	**duration [s]**	**frequency [Hz]**	**Δ distance [µm]**	**amplitude [µm]**	**data interval [ms]**	**autom. readjustment**	
**pump off**	-	1	-	-	20	-	
**linear ramp**	5	1	50	-	1000	-	
**sine function**	180	0	-	50	1000	on	
**linear ramp**	5	1	-50	-	1000	-	
	2	-	-	-	-	-	
**pum on**	2	-	-	-	-	-	
**linear ramp**	5	1	100	-	20	-	
**linear ramp**	5	1	160	-	1000	-	
**sine function**	7200	1	-	160	1000	-	
**pump off**	2	-	-	-	-	-	
**linear ramp**	5	1	-210	-	1000	-	
**sine function**	180	0	-	50	1000	on	
**linear ramp**	5	1	210	-	1000	-	
	2	-	-	-	-	-	
**pump on**	2	-	-	-	-	-	
**sine**	7200	1	-	160	1000	-	
**pump off**	2	-	-	-	-	-	
**linear ramp**	5	1	-210	-	1000	-	
**sine function**	180	0	-	50	1000	on	
**linear ramp**	5	1	210	-	1000	-	
	2	-	-	-	-	-	
**pump on**	2	-	-	-	-	-	}
**sine**	14400	1	-	160	1000	-	}
**pump off**	2	-	-	-	-	-	}
**linear ramp**	5	1	-210	-	1000	-	} 5 x
**sine function**	180	0	-	50	1000	on	}
**linear ramp**	5	1	210	-	1000	-	}
	2	-	-	-	-	-	}
