# Supplementary material for: BMP2 and mechanical loading cooperatively regulate immediate early signalling events in the BMP pathway
Source: BMC Biol. 2012 Apr 30;10:37. doi: 10.1186/1741-7007-10-37 (PMC3361481; doi:10.1186/1741-7007-10-37)
Supplement: Additional file 5 — Primer sequences. Sequence of primers used for qRT-PCR. [file 1741-7007-10-37-S5.PDF]

Gene	Primer sequence 5'-3'	Accession number
**HRPT**	fw- CTTTGCTGACCTGCTGGATT	NM_000194
	rev- CTGCATTGTTTTGCCAGTGT	
**Runx2**	fw- CAGGCAGGTGCTTCAGAACT	NM_001024630
	rev- GGCGGGGTGTAAGTAAAGGT	
**c-fos**	fw- CAAGCGGAGACAGACCAACT	NM_005252
	rev- AGATCAAGGGAAGCCACAGA	
**osteopontin**	fw- CAGAATGCTGTGTCCTCTGAA	NM_001040058
	rev- GTCAATGGAGTCCTGGCTGT	
**Smad1**	fw- CAACAGAGGAGATGTTCAGGC	NM_005900
	rev- AGTGAAACCATCCACCAACAC	
**Smad5**	fw- GGATGAGTTTTGTCAAGGGTTG	NM_005903
	rev- ACAGAAGATATGGGGTTCAGAGG	
**Smad4**	fw- AAGGTCTTTGATTTGCGTCA	NM_005359
	rev- GTCCCCAGCCTTTCACAA	
**BMP2**	fw- CATGCCATTGTTCAGACGTT	NM_001200
	rev- CAACTGGGGTGGGGTTTT	
**BMP4**	fw- CCACGAAGAACATCTGGAGAAC	NM_001202
	rev- ATACGGTGGAAGCCCCTTT	
**BMP6**	fw- GCAGACCTTGGTTCACCTTATG	NM_001718
	rev- AGAATGTGTGTCCCCAGCA	
**BMP7**	fw-GTCAGGAGTTCGAGACCAGC	NM_001719
	rev-ATCTTGGCTCACTGCAACCT	
**Noggin**	fw- GTGCAAGCCGTCCAAGTC	NM_005450
	rev- GCTAGAGGGTGGTGGAACTG	
**Id1**	fw- GCTGCTCTACGACATGAACG	NM_002165
	rev- CCAACTGAAGGTCCCTGATG	
**Id2**	fw- GTGGCTGAATAAGCGGTGTT	NM_002166
	rev- TGTCCTCCTTGTGAAATGGTT	
**Dlx2**	fw- GGCGTTTCCAAAAGACTCAA	NM_004405
	rev- CGAAGCACAAGGTGGAGAAG	
**Dlx3**	fw-TTGTGCCTTTGGGAGTAAGG	NM_005220
	rev-GGCTGCTTCTCTCTGTTGCT	
**Dlx5**	fw- CCAGAGAAAGAAGTGACCGAG	NM_005221
	rev- CACCTGTGTTTGTGTCAATCC	
