# Supplementary material for: BMP2 and mechanical loading cooperatively regulate immediate early signalling events in the BMP pathway
Source: BMC Biol. 2012 Apr 30;10:37. doi: 10.1186/1741-7007-10-37 (PMC3361481; doi:10.1186/1741-7007-10-37)
Supplement: Additional file 6 — Validation of a reference gene for qRT-PCR. Validation of HPRT as house-keeping reference gene using geNorm software [69]. [file 1741-7007-10-37-S6.PDF]

	HPRT	GAPDH	EF1	actin	Normalisation Factor
**control**	1,01E+00	1,02E+00	1,00E+00	1,00E+00	**0,9939**
**load**	1,00E+00	1,00E+00	1,02E+00	1,00E+00	**0,9906**
**BMP2**	1,00E+00	1,05E+00	1,03E+00	1,04E+00	**1,0160**
**load+BMP2**	1,00E+00	1,02E+00	1,03E+00	1,00E+00	**0,9997**
	1,003742797	1,023047849	1,019378753	1,010686853	
**M < 1.5**	**0,029**	**0,023**	**0,024**	**0,020**	
